# Adapting a Behavioral Intervention for Caregivers of Children with Down Syndrome or Fragile X Syndrome: A Pilot Study of RUBI-DD

**DOI:** 10.3390/bs16030472

**Published:** 2026-03-22

**Authors:** Allison D. Blackburn, Walker McKinney, Allison M. Birnschein, Anna J. Esbensen, Shelley McKinley, Hilary Rosselot, Emily K. Hoffman, Craig Erickson, Rebecca Shaffer

**Affiliations:** 1 Division of Behavioral Medicine and Clinical Psychology, Cincinnati Children’s Hospital Medical Center, Cincinnati, OH 45229, USA; allison.blackburn@cchmc.org (A.D.B.); allison.birnscheinbarlow@cchmc.org (A.M.B.); 2 Department of Pediatrics, University of Cincinnati College of Medicine, Cincinnati, OH 45219, USA; anna.esbensen@cchmc.org (A.J.E.); craig.erickson@cchmc.org (C.E.); 3 Division of Developmental and Behavioral Health, Children’s Mercy Hospital, Kansas City, MO 64108, USA; wsmckinney@cmh.edu; 4 Department of Pediatrics, University of Missouri—Kansas City, Kansas City, MO 64108, USA; 5 Division of Developmental and Behavioral Pediatrics, Cincinnati Children’s Hospital Medical Center, Cincinnati, OH 45229, USA; emily.hoffman1@cchmc.org; 6 Division of Child and Adolescent Psychiatry, Cincinnati Children’s Hospital Medical Center, Cincinnati, OH 45229, USA; shelley.mckinley@cchmc.org; 7 National Fragile X Foundation, Washington, DC 20005, USA; hilary@fragilex.org

**Keywords:** Fragile X Syndrome, Down Syndrome, caregiver training, caregiver coaching, behavioral intervention, irritability, behaviors, caregiver-mediated intervention

## Abstract

Challenging behaviors, including noncompliance, aggression, hyperactivity, and impulsivity, are common among individuals with Fragile X Syndrome (FXS) and Down Syndrome (DS). To identify treatment needs specific to these populations, we conducted focus groups with caregivers and educators and used their input to adapt an evidence-based caregiver training program originally designed for caregivers of autistic children (i.e., The Research Units in Behavioral Intervention; RUBI). We then completed a feasibility trial in which five families of children with FXS and four families of children with DS completed a nine-session caregiver training program targeting behavioral principles, syndrome-specific information, and visual supports tailored to the unique needs of FXS or DS (adapted version of RUBI for non-autism developmental disabilities; RUBI-DD). The program demonstrated strong acceptability, with high caregiver satisfaction, 100% retention, and 100% session attendance. Across the combined sample, caregiver reports indicated significant improvements in irritability/aggression (*F*_(2,15.14)_ = 4.42, *p* = 0.03), lethargy/social withdrawal (*F*_(2,14.47)_ = 3.97, *p* = 0.04), stereotypies (*F*_(2,15.29)_ = 4.45, *p* = 0.03), hyperactivity (*F*_(2,15.14)_ = 6.51, *p* = 0.009), social inflexibility (*F*_(2,15.43)_ = 6.33, *p* = 0.01), demand-based noncompliance (*F*_(2,15.41)_ = 4.95, *p* = 0.02), and the impact of behavior on the family (*F*_(2,15.07)_ = 4.23, *p* = 0.04) following participation in RUBI-DD. Caregivers of children with FXS reported significant reductions in lethargy/social withdrawal (*F*_(2,8.000)_ = 6.256, *p* = 0.023) and hyperactivity (*F*_(2,8.000)_ = 12.497, *p* = 0.003) immediately post-treatment and upon 12-week follow-up (*g* = 1.153, *p* = 0.044, and *g* = 1.178, *p* = 0.003, respectively). Among families of children with DS, caregivers reported reductions in irritability and aggression (*F*_(2,5.047)_ = 14.073, *p* = 0.009) and improvements in the impact on the family (*F*_(2,6.000)_ = 5.489, *p* = 0.044) immediately post-treatment and at follow-up (*g* = 1.643, *p* = 0.016, and *g* = 0.448, *p* = 0.045, respectively). These findings support the feasibility, acceptability, and preliminary efficacy of RUBI-DD for children with FXS or DS.

## 1. Introduction

Challenging behaviors are common among children with intellectual disability (ID), and this population is twice as likely, compared to typically developing children, to have difficulties with inattention, impulsivity, and mood disturbances (Buckley et al., 2020; E. M. Dykens, 2007; Rojahn & Meier, 2009; Staunton et al., 2023; Sutherland et al., 2025). However, among children with ID, there is heterogeneity in behavioral concerns, in part related to etiologic heterogeneity. Fragile X syndrome (FXS) is the most common inherited cause (i.e., the genetic mutation is passed down from a biological parent) of ID (1/7000 males and 1/11,000 females (Hunter et al., 2014)), and Down Syndrome (DS) is the most common genetic cause (i.e., random cell error leads to an extra copy of chromosome 21) of ID (1/824 of live births (de Graaf et al., 2017). More than 75% of children with FXS exhibit challenging behaviors, including self-injury, aggression, hyperactivity, impulsivity, social skill difficulties, and noncompliance (Genovese & Butler, 2025; Hagerman et al., 2009; S. Hall et al., 2016; Muller et al., 2019). Children with DS also exhibit difficulties with noncompliance, aggression, hyperactivity, and impulsivity, yet also exhibit elopement and inattention (E. M. Dykens & Kasari, 1997; L. Patel et al., 2018; Yahia et al., 2014). Rates of challenging behaviors in children with DS range from 18 to 43% (Capone et al., 2006; Corrice & Glidden, 2009; E. M. Dykens & Kasari, 1997; E. Dykens et al., 2015; L. Patel et al., 2018; Visootsak & Sherman, 2007) and are elevated relative to typically developing children matched in mental age (Capone et al., 2006; Coe et al., 1999; Cuskelly & Dadds, 1992; Evans et al., 2005; Evans & Gray, 2000; Feeley & Jones, 2008; Foley et al., 2015; Gath & Gumley, 1986; Glenn & Cunningham, 2007; Loveland & Kelley, 1991; Nicham et al., 2003; Pitcairn & Wishart, 1994; Pueschel et al., 1991; van Gameren-Oosterom et al., 2011). Challenging behaviors dramatically limit the daily functioning of children with FXS or DS, adversely impact their social–emotional and self-regulation skills, place significant burden on caregivers, negatively impact the child and family’s quality of life, and contribute to parental stress (Abbeduto et al., 2004; Bailey et al., 2000; Esbensen & Seltzer, 2011; Fielding-Gebhardt et al., 2020; Fuca et al., 2022; Hauser et al., 2014; Most et al., 2006; Owen et al., 2004; Roach et al., 1999; Sanders & Morgan, 1997; Will et al., 2016). Caregivers of children with FXS or DS report higher rates of stress, depressed mood, and pessimism, as well as lower rates of well-being and marital satisfaction in comparison to caregivers of typically developing children (Abbeduto et al., 2004; Deutsch et al., 2008; Ekstein et al., 2011; Fidler et al., 2000; Hauser-Cram et al., 2001; Hauser et al., 2014; Most et al., 2006; Roach et al., 1999; Roberts et al., 2009; Sanders & Morgan, 1997; Sarimski, 1997; Scott et al., 1997; Skotko et al., 2011). This demonstrates that, despite being different disorders, there is overlap between FXS and DS child behavior and caregiver stress profiles.

These behavioral and family profiles highlight the importance of interventions to address challenging behaviors, but barriers, including the interpretation of those behaviors and access to evidence-based interventions, persist for these combined groups. Caregivers’ and educators’ interpretations of the causes of the challenging behaviors may prevent children with DS or FXS from receiving behavioral treatment. In one study, mothers of children with DS or other intellectual disabilities interpreted noncompliant child behaviors as temporary and normal (Ly & Hodapp, 2002). Professionals and parents may also erroneously attribute challenging behaviors to the medical diagnosis of DS or FXS rather than mental health or environmental concerns that could be addressed via behavioral interventions (L. R. Patel et al., 2020). While it is true that core genetic and biological components of the diagnosis lead to behavioral difficulties, such as decreased Fragile X messenger ribonucleoprotein (FMRP) leading to increased sensitivity and stress, this does not mean that behavioral and prevention interventions will be unsuccessful in lowering challenging behaviors (S. S. Hall et al., 2020).

Additionally, despite federal prioritization of behavioral treatment development for FXS and DS (NICHD, 2009, 2014), research has heavily focused on pharmacological interventions for both populations, especially in FXS (Baumer & Capone, 2023; Hart et al., 2017; NICHD, 2009, 2014). Most non-pharmacological treatments recommended for challenging behaviors in FXS and DS have yet to be tested empirically against control groups in these populations. The lack of behavioral treatment trials that include children with FXS or DS significantly limits the ability of clinicians to generalize findings to these developmental syndromes and provide individualized care to these populations. Thus, there is a continued need to develop behavioral treatments that can improve daily functioning, decrease challenging behaviors, and reduce caregiver burden.

Despite the limited availability of well-established, evidence-based behavioral interventions for children with FXS or DS, small sample studies have demonstrated initial promise of function-based behavioral therapies, including functional communication training and applied behavior analysis (ABA; S. S. Hall et al., 2020). A review of the existing literature identified four components of behavioral interventions that have been used to treat challenging behaviors in individuals with DS: addressing setting events, antecedent (or prevention) strategies, skill-building strategies, and consequence-based strategies (Feeley & Jones, 2006). Consistent with this framework, a meta-analysis of single-case studies demonstrated that ABA-based interventions are effective in reducing challenging behavior in DS (Neil et al., 2021). Additionally, one feasibility study of a parent training program adapted for children with DS reported promising initial outcomes, further supporting the potential utility of caregiver-focused behavioral interventions in this population (Stone-Heaberlin et al., 2024).

For children with FXS and severe problem behavior, Kurtz et al. (2015) provided evidence of the efficacy of clinician-delivered, function-based behavioral intervention, reporting a mean decrease of 95.2% in problem behavior from baseline. Although this intervention was not caregiver-mediated, caregivers were successfully trained to high levels of procedural fidelity (≥90%). More recently, Monlux et al. (2019) demonstrated the efficacy and feasibility of delivering function-based, caregiver-mediated behavioral treatment via telehealth for children with FXS, representing an important step toward evaluating caregivers as primary interventionists in the treatment of severe problem behavior in FXS. Additional studies further support the promise of caregiver-mediated, function-based approaches, including reductions in specific challenging behaviors following brief telehealth caregiver education (Miranda et al., 2025) and caregiver-mediated functional communication training (S. S. Hall et al., 2020). Collectively, these findings provide strong preliminary evidence that function-based behavioral therapies, including those that actively involve caregivers, are both feasible and efficacious for children with FXS and DS and that caregiver inclusion may enhance the ecological validity of intervention delivery (Kurtz et al., 2015; Neil et al., 2021; Machalicek et al., 2014).

To develop and test more rigorous behavioral treatment trials conducted specifically in FXS and DS, it is necessary to examine evidence from efficacious treatment trials administered among children with related developmental disorders. In practice, treatment recommendations for challenging behavior in FXS and DS are largely derived from trials involving children with idiopathic autism spectrum disorder (ASD) or attention-deficit/hyperactivity disorder (ADHD; Davidson et al., 2022). Emerging evidence suggests that adapting caregiver-mediated interventions developed for ASD may be an appropriate and promising approach for children with DS and FXS (Vismara et al., 2019). One established behavioral treatment for children with ASD is a manualized caregiver training model, created by the Research Units on Behavioral Intervention Autism Network (RUBI; Bearss et al., 2018b). RUBI is a structured caregiver training program that teaches caregivers how to utilize the principles of applied behavioral analysis to decrease challenging behavior, increase positive behavior, and teach their child new skills. Through weekly one-on-one therapy sessions, caregivers learn behavioral principles including the antecedent–behavior–consequence model, functions of behavior, antecedent management strategies, and consequence-based strategies that focus on reducing challenging behaviors, including aggression, self-injury, frequent tantrums, and noncompliance. Each weekly session includes activity worksheets and video vignettes to enhance the caregiver’s learning and a homework assignment. The RUBI protocol includes 11 core sessions, one home visit, and supplemental (optional) sessions that are concern-specific (toileting, sleep, token economies, time-out, crisis management, imitation skills, and mealtime behaviors). Randomized controlled trials of RUBI demonstrated the efficacy of this program for young children with ASD (Aman et al., 2009; Bearss et al., 2015; Iadarola et al., 2018; Scahill et al., 2016), resulting in improvements in irritability, serious maladaptive behavior, disruptive behavior, and adaptive skills. Additionally, the program also drove improvements in self-reported parental competence and parental stress (Iadarola et al., 2018).

Although RUBI was developed for children with ASD, the function-based behavioral principles and caregiver-mediated delivery model are likely to be effective for treating challenging behaviors in other developmental disorders, including FXS and DS. Caregiver-mediated behavioral interventions have a solid evidence base for treating disruptive behaviors across diagnostic groups, supporting their transdiagnostic utility (Kazdin, 2005). Children with DS and FXS share several behavioral characteristics with autistic youth, including repetitive/stereotyped behaviors, oppositional behaviors, and communication impairments (Côté et al., 2020), while also frequently exhibiting challenging behaviors related to anxiety, inattention, or impulse control (S. Hall et al., 2016; Hustyi et al., 2014; Woodcock et al., 2009). Teaching caregivers to utilize functional behavioral assessment prior to intervention may be particularly valuable for these populations. This approach allows caregivers to identify maintaining variables, including setting events such as sleep disturbances or gastrointestinal discomfort, which are common in both FXS and DS and contribute to emotional dysregulation and challenging behaviors (Soltani et al., 2023). A caregiver-mediated model may be especially relevant for families of children with FXS, where hereditary patterns can uniquely influence caregiver stress and family functioning. Taken together, despite differences in syndrome-specific phenotypes, the shared behavioral characteristics of FXS and DS support the potential utility of a function-based core curriculum supplemented with targeted, syndrome-informed modules (Stein, 2016). Additionally, because challenging behaviors often persist into the school-age years for children with FXS and DS (S. Hall et al., 2016; Hustyi et al., 2014), developmentally flexible interventions are needed. However, before implementing an intervention developed for ASD in these populations, it is critical to incorporate stakeholder feedback to ensure relevance and acceptability. Caregiver perceptions of treatment procedures and goals (i.e., acceptability, relevance, effectiveness, demandingness) are closely linked to engagement, retention, and treatment outcomes, underscoring the importance of stakeholder-informed adaptation to enhance social validity (Kazdin, 2000; Butler & Titus, 2015).

Given that FXS and DS are the most prevalent genetic causes of intellectual disability, both associated with high rates of challenging behavior, caregiver stress, and limited evidence-based behavioral interventions, including both FXS and DS allowed for evaluation of an adapted intervention across and within disorders. This combined approach also facilitated recruitment and capitalized on similarities across the behavioral profiles of children with FXS or DS. Accordingly, we piloted an adapted version of the RUBI program (RUBI-DD) to assess its feasibility and acceptability in these populations and to examine its applicability to non-autism developmental disabilities. First, we conducted focus groups with caregivers of children and adolescents with FXS or DS. We also included educators with experience working with these two populations. Next, we adapted the RUBI curriculum and created new syndrome-specific material based on the feedback received in the focus groups. Finally, we piloted the new curriculum with a small group of families with children diagnosed with FXS or DS. We hypothesized that the adapted curriculum would be feasible and acceptable and would lead to improved irritability, noncompliance, caregiver stress, and caregiver perceptions of the impact of the child’s health condition. We had three research questions: First, is the RUBI program applicable to families of children with FXS and DS? If so, how may the program need to be adapted to better fit the families’ behavioral concerns? Second, is the RUBI-DD program feasible and acceptable to implement with families of children with FXS and DS? Third, does the RUBI-DD program result in decreased behavioral concerns and caregiver stress? The final research question is exploratory in nature, given that this was a pilot study with a small sample.

## 2. Materials and Methods

### 2.1. Intervention Development and Description

Focus groups were conducted with families of children with FXS or DS and educators who work with these two populations. Participants were recruited to the focus groups from clinics at Cincinnati Children’s Hospital Medical Center, including the Cincinnati Fragile X Research and Treatment Center and the Thomas Center for Down Syndrome. Flyers were distributed across each of these locations advertising the study. In addition, community groups including the Down Syndrome Association of Greater Cincinnati and the Tri-State Fragile X Alliance publicized the focus groups through social media and their mailing lists. Focus groups were presented with information via PowerPoint about each session of the original RUBI curriculum, and they provided input as to how helpful each topic would be or would have been in the past for their family or patients. The focus groups were facilitated by two licensed psychologists with expertise in FXS and DS. Detailed field notes were collected, and audio was recorded for each focus group. Each focus group lasted approximately 75 min. Children of participating families ranged from 6 to 20 years of age. There were 8 FXS families, 6 DS families, and 2 special education teachers who participated across 3 focus groups (5–6 in each group). Demographics were not collected from the focus group participants. The focus group feedback was compiled for each session across groups and the facilitators identified areas for adaptation. Agreement was obtained between the two leaders about the adaptations before they were conducted.

Focus group participant feedback indicated significant interest in the behavioral interventions presented in the RUBI curriculum as well as several additional topics (e.g., special education information, psychoeducation about the syndrome). The families also indicated that some sessions did not apply to their child and were not needed. Accordingly, the curriculum was condensed, and sessions on topics of interest to the families were added. Sessions related to functional communication training, daily schedules, and teaching skills were removed. Sessions related to syndrome-specific content (i.e., common characteristics, phenotypes, associated health conditions, support resources), evidence-based treatment options, and special education were added. Where topics remained the same, syndrome-specific content was added and vignettes were modified to be inclusive of common concerns. For example, the session related to sleep concerns was modified to include information about the co-occurrence of sleep apnea in DS and the most common sleep disturbances in children with FXS.

The outline of the curriculum, an adapted version of RUBI for non-autism developmental disabilities (RUBI-DD), based on both caregiver and professional input, is presented below in Table 1. We tailored our adapted curriculum to account for the unique cognitive profiles of children with FXS and DS, including more pronounced processing speed impairments (Dehkharghani, 2025; Kaat et al., 2022). Specifically, to leverage the relative strengths in visual memory and nonverbal skills for these populations, our curriculum highlighted the use of verbal prompting with modeling, visuals, social stories, transition cues, and visual schedules (E. Dykens et al., 2000).

### 2.2. Pilot Study of RUBI-DD

To pilot the newly adapted program, families were recruited via direct referral from community physicians and clinics at Cincinnati Children’s Hospital Medical Center, including the Cincinnati Fragile X Research and Treatment Center and the Thomas Center for Down Syndrome. Flyers were distributed across each of these locations advertising the study. In addition, community groups including the Down Syndrome Association of Greater Cincinnati and the Tri-State Fragile X Alliance publicized the study through social media and their mailing lists. Inclusion criteria for the study were similar to those of the original RUBI program (Bearss et al., 2015), including (a) documented diagnosis of an FMR1 full mutation (i.e., FXS) or Trisomy 21 (i.e., DS); (b) between the ages of 3–12 years old; (c) English as the primary spoken language in the home; (d) receptive language of at least 18 months as determined by the Differential Abilities Scale—2nd Edition; (e) irritability scores of 6 or more or hyperactivity score of 12 or more on the Aberrant Behavior Checklist—Community (ABC). Traditional scoring of the ABC was used for the combined sample, but for FXS-specific analyses, the Fragile X Syndrome scoring was utilized. These scores are the mean ABC scores for children with DS from previous studies (Stores et al., 1998). Participants were excluded from the study if they had deafness or blindness that would interfere with the valid administration of study measures, or if they had a medication change within the last two months. Initially, a randomized controlled trial was planned for this study, but it was discontinued due to recruitment challenges. After screening, 9 children enrolled in the caregiver training intervention (5 FXS and 4 DS). Clinical characteristics of the study participants are reported in Table 2 for the 9 children who received the intervention. All caregiver participants were women, with 8 mothers and 1 grandmother with custody of the child.

### 2.3. Pilot Intervention

RUBI-DD is a standardized eight-session caregiver training intervention with an additional optional session chosen by the family from three possible options (Table 1). RUBI-DD was designed to be implemented through one-hour weekly individual sessions without the child present. Key behavioral techniques include reinforcement and contingency management systems, exposure and extinction, stimulus control, prevention strategies, and informational sessions about education and appropriate treatments. These principles have each been successfully used to treat common challenging behaviors among children with developmental disabilities during individually tailored treatments (Richdale & Wiggs, 2005). Each session included direct instruction, vignettes, activity sheets, role play, and modeling to promote skill acquisition. Caregivers were provided with data collection assignments to encourage the implementation of skills at home after each session. All sessions were facilitated by a licensed psychologist with experience conducting interventions with both populations.

### 2.4. Therapist Training

The senior author, a licensed psychologist with experience facilitating interventions for both children with FXS and DS, served as the therapist delivering the intervention. She was previously trained as a therapist in RUBI, including review of her videos by an expert RUBI clinician. Treatment fidelity checklists were completed by the therapist at the end of each session to ensure coverage of pre-determined key session topics and implementation of session goals and objectives. The fidelity sheets were utilized from the original RUBI validation study and slightly adapted for added content. Session integrity was measured by rating each session goal on a three-point scale (0 = goal was not introduced or covered by the clinician, 1 = goal was partially achieved, 2 = goal was fully achieved). Caregiver adherence was measured by rating the degree to which the caregiver participated, responded correctly, and completed activities on a three-point scale (0 = caregiver did not demonstrate skill or understanding/did not complete assignments, 1 = caregiver understood or responded correctly to a few of the queries/partially completed assignment, 2 = caregiver understood and responded correctly to nearly all queries/completed all assignments).

### 2.5. Descriptive Assessments

Families participated in a baseline visit as a caregiver–child dyad to confirm eligibility (receptive language of at least 18 months) and to understand the family’s functioning at baseline. The baseline visit took approximately 60–90 min and included cognitive and adaptive assessment of the child, caregiver-reported outcome measures, and a clinician rating of the severity of impairment. The Differential Abilities Scale-II (DAS-II; Elliot, 2007) was administered by clinical psychologists who specialized in FXS or DS. The DAS-II is a measure of cognitive abilities appropriate for individuals aged 2–18 years and recommended for studies of cognition among individuals with ID (Elliot, 2007). A trained study coordinator completed the Comprehensive Parent Interview form of the Vineland-3 to assess adaptive behavior skills. The Clinical Global Impressions Scale-Severity (CGI-S; Guy, 1976) was utilized as a clinician-rated measure to assess overall severity of impairment based on caregiver qualitative reports of behavior and the clinician’s total clinical experience with the relevant population. The CGI-S has been used widely in ASD pharmacology and behavioral trials (Bearss et al., 2015; Minshawi et al., 2016). It provides a qualitative measure of global illness severity through a rating from 1 to 7 (1 = normal, not at all ill; 2 = borderline ill; 3 = mildly ill; 4 = moderately ill; 5 = markedly ill; 6 = severely ill; 7 = among the most extremely ill patients).

### 2.6. Outcome Measures

Caregiver-reported outcome measures were completed at baseline, one-week post- treatment and 12 weeks post-treatment. Follow-up visits also took approximately 60–90 min and were completed as caregiver–child dyads. The Aberrant Behavior Checklist (ABC; Aman & Singh, 1986) was used to assess symptoms of child problem behaviors. The ABC is a 58-item caregiver-completed rating scale composed of 5 subscales: lethargy/social withdrawal (16 items), irritability (15 items), inappropriate speech (4 items), hyperactivity (16 items), and stereotypy (7 items). Caregivers completed the ABC at baseline, at the end of treatment, and again at 12 weeks follow-up. Response options ranged from 0 “no problem” to 3 “severe problem.” Scores for each subscale were summed. In other studies, 25% change has been used as a benchmark of clinically meaningful change (McCracken et al., 2002). The ABC irritability scale was used in the original RUBI trial. For the FXS subsample analyses, FXS-specific validated scoring of the ABC (ABC-FXS) was utilized.

The Home Situations Questionnaire—ASD (Chowdhury et al., 2016; HSQ-ASD) was used to assess noncompliance. The version of the HSQ used in this study came from a previous RUBI study that included more items about demand-based noncompliance. The HSQ-ASD has two subscales (12-items each) with good internal consistency: Socially Inflexible (α = 0.84) and Demand-Specific (α = 0.89; Chowdhury et al., 2016).

Parental stress was measured with the Parental Stress Index—4th Edition (PSI; Abidin, 2012). The PSI is commonly used for evaluating the magnitude of stress in the caregiver–child system (Abidin, 2012). It measures three domains of stress, including child characteristics, caregiver characteristics, and situational/demographic life stress. Internal consistency is high (α = 0.75–0.87) and test–retest reliability is satisfactory (0.70; Abidin, 2012).

The Family Impact Questionnaire (FIQ; Donenberg & Baker, 1993) measures caregiver perceptions of how their child’s health condition impacts their family. Internal consistency was good to excellent within a previous sample (alphas > 0.81; Baker et al., 2003). For this study, we only examined the Positive Parent and Total Negative Impact subscales, as was previously utilized in a RUBI for DS study (Stone-Heaberlin et al., 2024).

Quality of life was also measured with the Pediatric Quality of Life Family Impact Module (PedsQL FIM; Varni et al., 2004), measuring the impact of child chronic health conditions on self-reported parental physical, emotional, social, and cognitive functioning; communication; and worry. It also measures caregiver-reported family functioning in the form of daily activities and family relationships. Caregivers rate 36 items on a 5-point Likert scale (0 = never a problem; 4 = always a problem) with higher scores indicating more challenges or poor quality of life. Internal consistency for all the PedsQL FIM subscales is high. For this study, the Health-Related (α = 0.94), Family (α = 0.92), and Total Impact (α = 0.96) scales were examined (Medrano et al., 2013; Varni et al., 2004).

The Clinician Global Impressions—Improvement (CGI-I; Guy, 1976) was utilized as a clinician-rated outcome measure to assess response to treatment since baseline, specifically as it relates to challenging behaviors. Although they are both Clinical Global Impressions, the CGI-S is a rating of overall severity (Descriptive Measure) and the CGI-I is a rating for measuring improvement (outcome measure). A trained, independent clinician rated the CGI-I based on qualitative information about behavior change provided by caregivers at each follow-up visit. The clinician was trained with gold standard vignettes and was blinded to the condition. The CGI-I provides a qualitative measure of treatment response through a rating from 1 to 7 (1 = very much improved; 2 = much improved; 3 = minimally improved; 4 = no change; 5 = minimally worse; 6 = much worse; 7 = very much worse). Ratings of “very much improved” indicate positive behavior changes in multiple settings, whereas “much improved” may indicate improvement that is marked in one setting more than others, or approximately 50–70% improvement. “Minimally improved” ratings reflect slight changes in one environment, or approximately 25% improvement.

### 2.7. Acceptability

Caregivers provided feedback using the Parent Consumer Satisfaction Questionnaire (PCSQ; Ollendick et al., 2016) following the intervention. The PCSQ measures feelings toward the overall program content, the format of the program, activities included in the program, and interactions with the therapist(s) on a 7-point Likert scale, with higher scores indicating greater satisfaction.

### 2.8. Data Analysis

Descriptive statistics were used to examine acceptability and feasibility variables, including caregiver satisfaction, fidelity, and study attrition. For treatment outcomes, we analyzed the combined DS+FXS group as well as each DS and FXS diagnostic group separately (i.e., three sets of analyses). Individual linear mixed effects models examined the impact of treatment on each dependent outcome, including challenging behaviors from the ABC, noncompliance from the HSQ, caregiver quality of life from the PedsQL, and caregiver stress from the PSI and the impact on the family via FIQ. Visit type (baseline vs. post-treatment vs. 12 weeks post-treatment) was the fixed effect, and subject/participant was the random effect in all models. For all models, the normality of residuals was assessed and confirmed using visual inspection of Q–Q plots. Significant effects of treatment were followed up with post hoc paired t-tests (baseline vs. post-treatment; baseline vs. 12 weeks post-treatment; post-treatment vs. 12 weeks post-treatment). Effect sizes (Hedge’s *g*) are also reported to help contextualize findings and aid power analyses used to estimate needed sample sizes for future studies. Hedge’s *g* effect sizes are interpreted in the same way as Cohen’s *d*, where values of 0.2, 0.5, and 0.8 correspond to small, medium, and large effects, respectively (Cohen, 1992).

## 3. Results

### 3.1. Caregiver Satisfaction

On a 7-point Likert scale where higher scores are better, families reported that they would recommend the program to others (M = 6.56) and that they had positive feelings toward the program (M = 6.11). In terms of verbal information presented in the program, families reported it was somewhat easy to understand (M = 5.33) and contained useful information (M = 6.11). In terms of the homework in the program, families reported it was somewhat easy to understand (M = 5.22) and useful (M = 6.11). For the written material provided in the sessions, families reported it was somewhat easy to understand (M = 5.89) and useful (M = 6.11). In terms of the therapist, families reported high satisfaction with the therapist’s teaching (M = 6), that the therapist was highly prepared (M = 6.11), that they were satisfied with the therapist’s concern for the caregiver and child (M = 6.56), that the therapist was helpful (M = 6.44), and that they personally liked the therapist (M = 6.33).

### 3.2. Fidelity

Similar to the original RUBI trial, fidelity was rated by the therapist at the end of each session for the therapist’s adherence to the material and the caregiver’s adherence to the session expectations, such as applying the material in session and homework completion. Therapist-rated fidelity to the session material averaged 99% and therapist-rated caregiver adherence also averaged 99%. Despite strong recruitment histories for both populations at Cincinnati Children’s Hospital Medical Center, recruitment was challenging given the required in-person appointments for weekly therapy. Many eligible families (65% of families contacted) were unable to travel to the hospital often enough for the intervention despite a high level of initial interest in the study. However, there was 100% retention of all study participants and 100% attendance across participants.

### 3.3. Combined FXS and Down Syndrome

**Figure 1:**
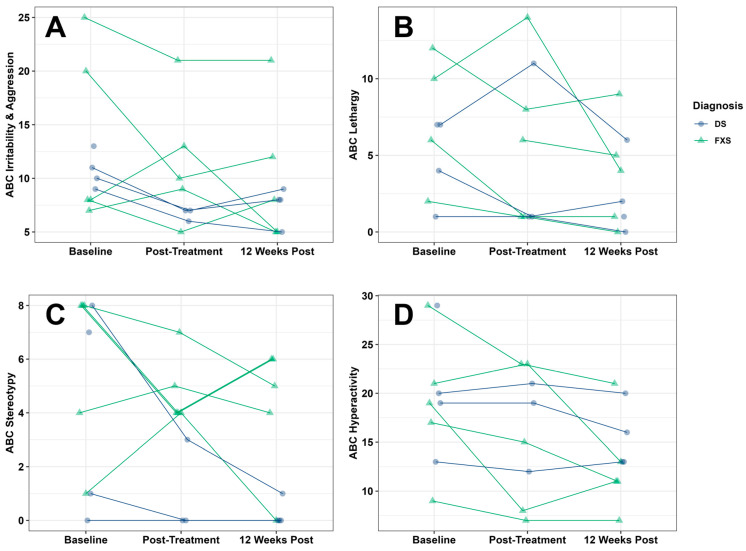
All four subfigures depict treatment-related improvements in challenging behaviors across diagnostic groups, as measured by the ABC. (**A**) Child irritability and aggression. (**B**) Child lethargy/social withdrawal. (**C**) Child stereotypies. (**D**) Child hyperactivity. Datapoints without longitudinal lines reflect missing data either due to (1) the caregiver of a participant with DS who did not complete the ABC immediately post-treatment or (2) several caregivers who omitted questionnaire items that prevented the scoring of an individual subscale.

**Figure 2:**
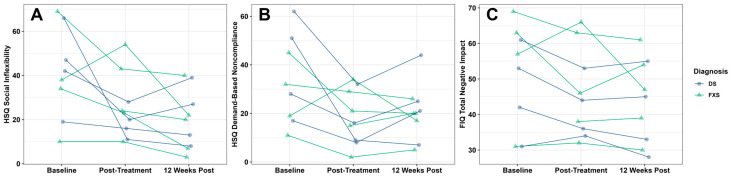
The three subfigures depict treatment-related improvements in challenging behaviors across diagnostic groups, as measured by the HSQ-ASD and FIQ. (**A**) Child social inflexibility. (**B**) Child noncompliance. (**C**) Negative impact on family. Datapoints without longitudinal lines reflect missing data either due to (1) the caregiver of a participant with DS who did not complete the HSQ-ASD or FIQ immediately post-treatment or (2) several caregivers who omitted questionnaire items that prevented the scoring of an individual subscale.

**Figure 3:**
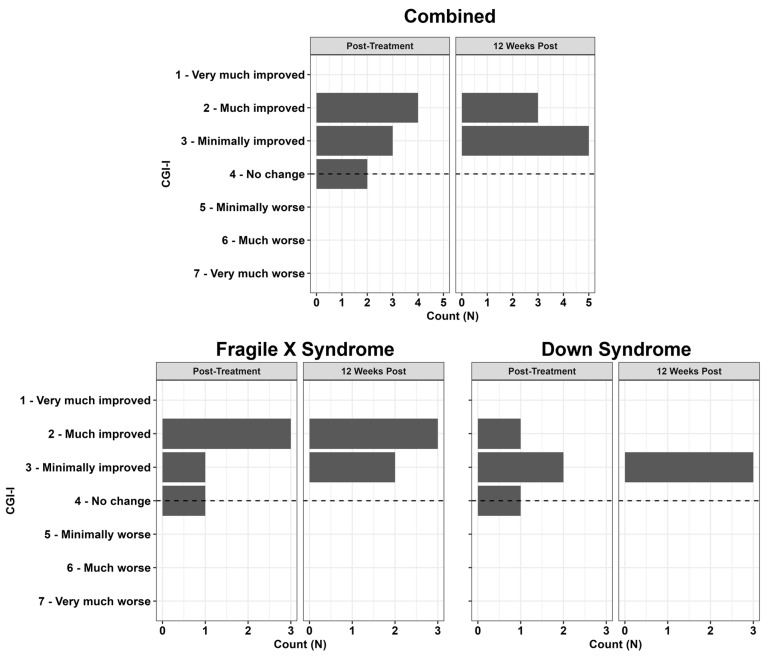
The three subfigures depict treatment-related improvements as rated by clinicians on the CGI-I. Combined depicts the CGI-I improvements of the combined FXS and DS sample. Fragile X Syndrome depicts the CGI-I improvements of the FXS sample. Down Syndrome depicts the CGI-I improvements of the DS sample.

Clinical scores at baseline, one week post-treatment, and 12 weeks post-treatment are presented in Table 3. In our combined FXS and DS sample (*N* = 9), RUBI-DD was associated with improvements in child irritability/aggression (Figure 1A; ABC Subscale I: *F*_(2,15.14)_ = 4.42, *p* = 0.03), lethargy/social withdrawal (Figure 1B; ABC Subscale II: *F*_(2,14.47)_ = 3.97, *p* = 0.04), stereotypies (Figure 1C; ABC Subscale III: *F*_(2,15.29)_ = 4.45, *p* = 0.03), hyperactivity (Figure 1D; ABC Subscale IV: *F*_(2,15.14)_ = 6.51, *p* = 0.009), social inflexibility (Figure 2A; HSQ Social Inflexibility subscale: *F*_(2,15.43)_ = 6.33, *p* = 0.01), and demand-based noncompliance (Figure 2B; HSQ Demand-Specific subscale: *F*_(2,15.41)_ = 4.95, *p* = 0.02). RUBI-DD was also associated with improvements in overall impact on the family as measured by the FIQ Total Negative Impact score (Figure 2C; FIQ Total Negative Impact: *F*_(2,15.07)_ = 4.23, *p* = 0.04). All other outcomes were non-significant (ABC: Subscale V; FIQ: Positive Parent subscale; PSI: Parental Distress subscale, Parent-Child Dysfunctional Interaction subscale, Difficult Child subscale, and total score; PedsQL FIM: Health-Related subscale, Family subscale, and total score). For all outcomes except the HSQ Demand-Specific subscale, non-significant treatment gains were observed immediately following treatment, and stronger, significant treatment gains were observed 12 weeks after treatment. For the HSQ Demand-Specific subscale, significant treatment gains were observed immediately following treatment, and marginally significant gains were observed 12 weeks after treatment (*p* = 0.059). Effect sizes and *p*-values for post hoc paired t-tests are reported in Table 4. For CGI-I ratings at the end of treatment, 44% were Much Improved, 33% Minimally Improved, and 22% had no change. At the 12-week follow-up visit, 37% were Much Improved and 63% Minimally Improved (Figure 3: Combined).

### 3.4. Fragile X Syndrome Group

In our FXS sample, RUBI-DD was associated with improvements in social unresponsiveness (Figure 1B; ABC FXS Subscale II: *F*_(2,8.000)_ = 6.256, *p* = 0.023) and hyperactivity (Figure 1D; ABC FXS Subscale IV: *F*_(2,8.000)_ = 12.497, *p* = 0.003). All other outcomes were non-significant (ABC: Subscales I, III, V, VI; HSQ Social Inflexibility and Demand-Specific subscales; FIQ Positive Parent and Total Negative Impact subscales; PSI Parental Distress subscale, Parent-Child Dysfunctional Interaction subscale, Difficult Child subscale, and total score; PedsQL FIM: Health-Related subscale, Family subscale, and total score). For both significant outcomes, post hoc paired t-tests indicated that significant treatment gains were observed both immediately after treatment and 12 weeks after treatment (Table 3). For CGI-I in the FXS group, following the end of treatment, 60% were Much Improved, 20% Minimally Improved, and 20% had no change. At the follow-up visit, 60% were Much Improved and 40% were Minimally Improved (Figure 3: Fragile X Syndrome).

### 3.5. Down Syndrome Group

In our DS sample, RUBI-DD was associated with improvements in child irritability/aggression (Figure 1A; ABC Subscale I: *F*_(2,5.047)_ = 14.073, *p* = 0.009). Post hoc paired t-tests indicated that significant treatment gains on the ABC irritability/aggression subscale I were observed both immediately after treatment and 12 weeks after treatment (Table 3). RUBI-DD was also associated with improvements in overall impact on the family (Figure 2C; FIQ Total Negative Impact: *F*_(2,6.000)_ = 5.489, *p* = 0.044). Post hoc paired *t*-tests indicated that non-significant treatment gains were observed immediately following treatment, and stronger, significant treatment gains were observed 12 weeks after treatment. All other outcomes were non-significant (ABC: Subscales II, III, IV, V; HSQ Social Inflexibility and Demand-Specific subscales; FIQ Positive Parent subscale; PSI Parental Distress subscale, Parent-Child Dysfunctional Interaction subscale, Difficult Child subscale, and total score; PedsQL FIM: Health-Related subscale, Family subscale, and total score). For CGI-I in the DS group at the end of treatment, 25% were Much Improved, 50% Minimally Improved, and 25% had no change. At follow-up, 100% were Minimally Improved (Figure 3: Down Syndrome).

## 4. Discussion

Despite elevated rates of challenging behaviors in children with FXS or DS, there are few evidence-based behavioral treatments tailored for these populations. To our knowledge, this pilot study is the first to adapt and test a comprehensive evidence-based caregiver-mediated program (RUBI) to address challenging behaviors among children with FXS or DS.

In this study, focus groups with caregivers of children with FXS and DS, as well as educators who work with these groups, identified elements of the existing RUBI curriculum that could be removed, condensed, or adapted to better fit the cognitive and behavioral profile of children with FXS or DS (Table 1). Specifically, caregivers provided examples of challenging behaviors that were commonly demonstrated by their children with FXS or DS but were not otherwise addressed in the original RUBI curriculum, such as increased anxiety and hyperexcitability. Few studies have systematically included the voice of caregivers in the development of an intervention, making these caregiver observations particularly informative. Within the focus groups, caregivers endorsed support for several RUBI-DD sessions to be expanded, including behavioral strategies such as stimulus control, differential reinforcement, and behavioral sleep strategies. They also provided feedback that it would be helpful to have sessions with education on the hereditary nature of FXS and family system impacts, education on selecting evidence-based treatments for DS and FXS, and guidance on educational supports applicable to older children. Additionally, caregivers hypothesized that their children may respond best to additional visual support strategies given their child’s unique visuo-spatial processing abilities.

Caregivers reported that RUBI-DD was highly acceptable. Specifically, caregivers who participated in the pilot implementation of the adapted intervention reported overall satisfaction with the program and shared that the program material and homework were understandable and useful. This is particularly meaningful feedback for RUBI-DD, considering that acceptability has not been consistently measured within caregiver-mediated intervention research. A scoping review of satisfaction in caregiver training programs found that only 5.5% of studies evaluated caregiver satisfaction data (Bjørknes & Ortiz-Barreda, 2021). RUBI-DD was also feasible, as evidenced by caregivers’ and therapists’ strong adherence to the planned program and their 100% attendance and retention rate. Attrition in this feasibility pilot was stronger than the original RUBI randomized control study (92%; Bearss et al., 2015). The small sample size of the current study likely accounts for this difference, but it is noteworthy that RUBI-DD was comprised of nine in-person appointments, compared to 12 in-person appointments and one home visit, as in the original RUBI protocol. These findings suggest that caregivers of children with DS or FXS are able and willing to implement a caregiver-mediated behavioral intervention.

In addition to this RUBI-DD pilot, demonstrating acceptability and feasibility, caregivers also reported significant reductions in challenging behaviors across children with FXS or DS. Both groups demonstrated meaningful improvements in irritability/aggression, lethargy/social withdrawal, stereotyped behaviors, hyperactivity, social inflexibility, and demand-specific noncompliance. Improvements in challenging behaviors are consistent with findings from studies of the original RUBI curriculum in ASD (Iadarola et al., 2018). Interestingly, many of the behavioral outcomes continued to strengthen over time, with the strongest improvements observed at 12 weeks post-treatment. This suggests that families continued to use the behavioral skills and strategies they learned beyond the treatment period to maintain improvements over time. The primary outcomes of this pilot demonstrate the value of tailoring evidence-based behavioral treatments for other developmental disorders. In addition to behavioral improvements, caregivers from both groups reported that their child’s behavior had a smaller negative impact on family life after the intervention, as measured by the FIQ Total Negative Impact scale. This outcome was also maintained over time. There were no observable differences in parenting stress or the impact of the child’s chronic health condition on caregiver or family functioning, as measured by the PedsQL FIM. It is possible that these represent broader constructs that are more resistant to treatment or that require long-term follow-up and generalization to drive meaningful change. Though challenging behaviors improve and have a smaller impact on the family, there are still many other medical complexities associated with both syndromes that continue to impact overall caregiver and family functioning.

Some areas of improvement were syndrome-specific when examining the FXS and DS groups separately. Specifically, significant reductions in lethargy/social withdrawal and hyperactivity were most evident within the FXS sample both immediately after and three months post-treatment. In contrast, children with DS had more significant reductions in irritability/aggression than children with FXS. Within our sample, the FXS group had higher rates of irritability/aggression at baseline compared to the DS group. This is consistent with existing behavioral phenotype comparisons demonstrating that children with FXS often exhibit higher rates of irritability, aggression, and externalizing behavior when compared to children with DS (E. M. Dykens et al., 2002; Symons et al., 2010). Thus, irritability may have been more responsive to change and it may have been easier to detect meaningful change in the DS group. Changes in stereotypies, social inflexibility, and demand-based noncompliance were found in the larger combined group, but not in the smaller syndrome-specific groups. Although it is possible there were selective improvements unique to either DS or FXS, we believe these syndrome-specific findings were driven by our limited statistical power to detect change, given that the syndrome-specific groups were small samples. 

In a small group of children with FXS or DS, the RUBI-DD program demonstrated meaningful improvements in behavior and the impact of behavior on family functioning. Improvements were maintained or improved over time, indicating that caregivers were able to continue using the strategies targeted in RUBI-DD after the active intervention. This study expands on findings from the only known previous feasibility pilot of a caregiver-mediated intervention for children with DS (Stone-Heaberlin et al., 2024) and supports the feasibility of RUBI-DD for both FXS and DS. Although both groups shared benefits, syndrome-specific patterns of behavioral change were also observed. Though further research is needed to best understand these differences, they highlight the importance of a flexible function-based behavioral curriculum with adaptations based on the unique behavioral profiles of the target population.

### Future Directions

The results of this feasibility study are promising, though limited by the small sample size and lack of a control group accounting for time and attention. Future research will need to extend these initial findings with a comparison or a control group. Another limitation of this study’s design is the lack of independent raters for measuring therapist fidelity. The RUBI-trained clinician who delivered the intervention rated each session for treatment fidelity using predetermined checklists. Future studies should obtain interrater reliability for a percentage of the sessions using raters who are not delivering the intervention. Finally, outcome measures relied only on caregiver-reported outcomes, and future studies will strengthen these findings by having direct behavioral data collection methods.

Recruitment efforts for this study were negatively impacted by the requirement for caregivers to attend weekly, in-person appointments in a clinical setting. Proximity to clinical centers with expertise in these genetic syndromes creates a barrier to families of children with FXS or DS. There are only 34 FXS treatment centers nationally, with many of them congregated in urban areas of the Midwest and Eastern regions, and the majority of those centers do not offer behavioral therapy. Specialty DS medical clinics are available in 40 states, but only two-thirds of these offer behavioral therapy. Thus, families travel long distances to access specialized behavioral services, which becomes burdensome when attending weekly sessions. Though limited, there is previous evidence supporting the effectiveness of behavioral interventions via telehealth in children with FXS (S. S. Hall et al., 2020, 2022). Future studies should incorporate telehealth delivery of RUBI-DD, with the aim of increasing access to specialized behavioral treatment for families of children with FXS or DS.

Telehealth platforms can connect caregivers and clinicians through web-conferencing, thus substantially reducing family burden by eliminating geographic barriers to specialized behavioral services. Further, telehealth significantly enhances attendance rates across healthcare settings, including behavioral services (Greenup & Best, 2025). In community settings, the RUBI caregiver training has been shown to be effective, acceptable, and feasible in a telehealth model of care for treating autism (Bearss et al., 2018a; Greathouse et al., 2025; Shanok et al., 2021). It is reasonable to assume that similar positive outcomes would be observed in the non-autism DD population using this modality. Future research may examine the effectiveness of RUBI-DD in FXS or DS when delivered in a telehealth model. When considering adapting RUBI-DD for a telehealth modality, adapting it to a group intervention is another consideration that should be piloted. There is strong feasibility and acceptability evidence for RUBI in a telehealth group format (Edwards et al., 2019; Jimenez-Gomez et al., 2023). Compared to individual behavioral interventions, group-based interventions can increase access to specialty care and have additional therapeutic benefits, such as increased social support from other caregivers (O’Donovan et al., 2019). Expanding RUBI-DD into these modalities would reduce significant barriers to accessing behavioral services for underserved populations.

It will also be important for future research to consider additional adaptations to increase the effectiveness of caregivers’ implementation of RUBI-DD skills and strategies at home. Potential supports include electronic access to materials, automated reminders to prompt skill-use during challenging times of the day, and joint caregiver–child sessions in which the therapist provides in vivo coaching. These adaptations may facilitate generalization of skills to the home as well as reduce executive functioning burdens on caregivers to remember the material and consistently apply the strategies. In fact, Cartwright and Mount (2022) found that adding a 15-min adjunctive parent support component to the RUBI protocol increased caregiver engagement, treatment integrity, and learned skills. By addressing acute caregiver stressors prior to the skill-training, caregiver stress was alleviated, allowing for increased focus and more effective learning.

An adjunctive caregiver support component is a particularly relevant consideration given that mothers of children with FXS often experience high levels of anxiety and executive functioning challenges (Bangert et al., 2021). Decreased caregiver stress is related to fewer behavioral concerns in typically developing children (Neece et al., 2012), highlighting the importance of addressing caregiver stress when addressing challenging behaviors in these populations. Our results show some promise in reducing caregiver perceptions of the impact of the child’s condition on the family (e.g., reduced total family negative impact), but not caregiver stress. Prior research has demonstrated that mindfulness and acceptance-based approaches are potential protective factors for mothers of children with FXS, and mindfulness interventions have been helpful in lowering anxiety for mothers with the FMR1 premutation (Wheeler et al., 2018). Additionally, children with FXS often experience high levels of anxiety and emotional dysregulation, which may also benefit from relaxation strategies (Shaffer et al., 2023). Future versions of RUBI-DD may benefit from the inclusion of brief relaxation or mindfulness strategies to be incorporated into each therapy session to support both the caregivers as well as their children. For example, each session could begin with a short, guided relaxation exercise, with caregivers encouraged to practice these strategies at home.

## 5. Conclusions

This is the first study to adapt RUBI, an established caregiver-mediated intervention for autism, to be utilized in FXS, and this is the second study of its adapted use in DS. Intervention content was adapted based on stakeholder focus group feedback. Overall, families found the intervention acceptable, were satisfied with the intervention, and improvements in challenging behaviors were found immediately following and three months post-treatment for both groups. Differences in behavioral changes between groups were observed but, due to limited statistical power, warrant further investigation. Future directions include the consideration of adjunctive components that specifically target caregiver stress, and a larger, randomized controlled trial with delivery of the intervention via telehealth.

## Figures and Tables

**Table 1:** FXS and DS RUBI-DD session overview.

	RUBI-DD Training Outline	Behavioral Principles	Adaptations Specific to DS and FXS
1	Introduction to the treatment program, an overview of common challenging behaviors, and the basic principles of the behavioral approach as it relates to addressing these difficulties	Data collection, function of behavior, antecedent–behavior–consequence model	Common challenging behaviors in DS and FXS reviewed as part of their behavioral phenotype and interwoven within session
2	Information about antecedent management (i.e., prevention strategies) commonly used to address behavior difficulties such as controlling the environment, changing the order of events, using visual or auditory cues, and addressing setting events	Prevention Strategies	Greater emphasis on visual supports and using a visual schedule as a strategy contingent upon visuo-spatial processing strengths. Given that the schedule session was removed, some of that content was included in this session
3	Information on types of reinforcers, how to identify reinforcers, the importance of recognizing the child being good, and stimulus control	Reinforcement & Stimulus Control	Greater emphasis on stimulus control (i.e., behavior is more likely to occur given the presence or absence of something) and differential reinforcement
4	Information about the rules of planned ignoring, modifications to planned ignoring, and common challenges when implementing	Planned Ignoring, Exposure & Extinction	Greater emphasis on extinction bursts. Specific to FXS, content was added about managing repetitive questions related to anxiety, including when to ignore versus not ignore
5	Information regarding steps for guided compliance along with a detailed plan of how to implement at home	Compliance Training	Greater emphasis on possible reasons for noncompliant behaviors common in DS and FXS, including slower processing speed. More practice opportunities provided for prompting
6	Information is provided regarding family support, impact of the syndrome on the family, and resources available at both the local and national levels	Syndrome-Specific Education	Unique to RUBI-DD, this session emphasized common characteristics, phenotypes, associated health conditions, and support resources for FXS or DS. For FXS, content included the hereditary nature of FXS and its impact on carriers and the larger family system
7	Family receives education about choosing appropriate treatment options and how to evaluate options utilizing available research and risk level involved. Common medication options are reviewed	Evidence-Based Treatment Education	Unique to RUBI-DD, emphasis on how to evaluate treatment research and warning about unfounded alternative treatment options. Emphasis also placed on medication given the high rate of medication treatment and pharmacological trials for FXS
8a	Optional: Token Economies are introduced. Likely more appropriate for school-age children	Reinforcement, Contingency Management Systems	This session was unchanged
8b	Optional: Sleep and common difficulties surrounding bedtime are addressed, as well as data tracking around sleep	Extinction & Reinforcement	Sleep problems are common in both FXS and DS. The session was tailored with education about the most common sleep challenges in both disorders and the impact of sleep disturbance on behaviors
8c	Optional: School information, including IEPs, IDEA laws, and ways to advocate for their child’s needs	School Services Education	Unique to RUBI-DD, this session emphasized common special education terms and procedures to enhance caregivers’ educational advocacy skills
9	Generalization and Maintenance are addressed with a final plan given to the family for exiting treatment	Generalization & Maintenance	This session was unchanged

**Table 2:** Clinical characteristics of study participants.

	Overall (*N* = 9)	Down Syndrome (*N* = 4)	Fragile X Syndrome (*N* = 5)
Age (years)	7.3 (3.1) Range: 3–12	7.0 (3.9) Range: 3–12	7.6 (2.7) Range: 5–12
% male	78% (*N* = 7)	75% (*N* = 3)	80% (*N* = 4)
Race & Ethnicity	11% African American 89% White 100% Non-Hispanic	100% White 100% Non-Hispanic	20% African American 80% White 100% Non-Hispanic
Caregiver % Female	100%	100%	100%
Full-Scale IQ	47 (10) Range: 36–63	54 (8) Range: 49–63	42 (9) Range: 36–55
Verbal IQ	46 (10) Range: 28–60	50 (9) Range: 39–60	43 (11) Range: 28–55
Nonverbal IQ	46 (16) Range: 26–76	49 (20) Range: 32–76	44 (15) Range: 26–67
Vineland Adaptive Behavior Composite	59 (15) Range: 31–87	69 (13) Range: 58–87	51 (12) Range: 31–60
Vineland Communication	49 (18) Range: 20–83	63 (15) Range: 46–83	38 (11) Range: 20–48
Vineland Socialization	64 (18) Range: 34–90	78 (11) Range: 66–90	54 (15) Range: 34–70
Vineland Daily Living Skills	62 (16) Range 38–98	69 (20) Range: 54–98	56 (11) Range: 38–67
CGI Severity Ratings			
4—Moderately Ill	55.6% (5)	75% (3)	40% (2)
5—Markedly Ill	44.4% (4)	25% (1)	60% (3)

Note. Values presented as Mean (SD) or as % (N); Abbreviations: IQ = Intelligence quotient; CGI = Clinical Global Impressions.

**Table 3:** Clinical scores at baseline, immediately post-treatment, and 12 weeks post-treatment.

Measure	Overall Mean (SD) (*N* = 9)			DS Mean (SD) (*N* = 4)			FXS Mean (SD) (*N* = 5)		
	Baseline	Post	12 Weeks Post	Baseline	Post	12 Weeks Post	Baseline	Post	12 Weeks Post
Aberrant Behavior Checklist (ABC)									
Irritability/Aggression	12.3 (6.2)	9.8 (5.2)	9.0 (5.0)	10.8 (1.7)	6.7 (0.6)	7.5 (1.7)	13.6 (8.3)	11.6 (6.0)	10.2 (6.7)
Lethargy/Social Withdrawal	6.1 (3.8)	5.4 (5.2)	3.1 (3.1)	4.8 (2.9)	4.3 (5.8)	2.2 (2.6)	7.5 (4.4)	6.0 (5.4)	3.8 (3.6)
Stereotypy	5.0 (3.5)	3.4 (2.4)	2.4 (2.7)	4.0 (4.1)	1.0 (1.7)	0.2 (0.5)	5.8 (3.2)	4.8 (1.3)	4.2 (2.5)
Hyperactivity	19.6 (6.5)	16.0 (6.5)	13.9 (4.5)	20.2 (6.6)	17.3 (4.7)	15.5 (3.3)	19.0 (7.2)	15.2 (7.8)	12.6 (5.2)
Inappropriate Speech	3.3 (1.9)	3.4 (2.0)	2.6 (1.7)	2.8 (2.1)	2.3 (0.6)	1.2 (1.0)	3.8 (1.8)	4.0 (2.3)	3.6 (1.5)
Aberrant Behavior Checklist (ABC)—Fragile X Subscales									
Irritability/Aggression	-	-	-	-	-	-	17.8 (10.7)	16.0 (7.1)	13.6 (9.3)
Socially Unresponsive	-	-	-	-	-	-	5.6 (2.3)	2.8 (0.8)	3.0 (1.7)
Stereotypy	-	-	-	-	-	-	5.6 (3.0)	4.6 (1.5)	4.2 (2.5)
Hyperactivity	-	-	-	-	-	-	13.0 (4.8)	9.8 (5.9)	7.6 (3.4)
Inappropriate Speech	-	-	-	-	-	-	3.8 (1.8)	4.0 (2.3)	3.6 (1.5)
Social Avoidance	-	-	-	-	-	-	4.0 (2.5)	4.0 (4.7)	2.4 (2.3)
Home Situations Questionnaire—ASD (HSQ)									
Social Inflexibility	40.6 (20.5)	25.4 (14.6)	19.9 (13.5)	43.5 (19.3)	18.8 (7.2)	21.8 (14.0)	37.8 (24.2)	30.8 (17.5)	18.4 (14.6)
Demand-Specific	33.1 (18.0)	18.4 (11.3)	20.6 (11.4)	39.5 (20.6)	16.2 (11.1)	24.2 (15.3)	26.8 (14.9)	20.2 (12.5)	17.6 (7.8)
Family Impact Questionnaire (FIQ)									
Positive Parent	20.6 (4.1)	20.7 (4.8)	20.9 (4.4)	20.2 (4.6)	20.0 (2.9)	20.8 (3.9)	21.0 (4.2)	21.2 (6.3)	21.0 (5.3)
Total Negative Impact	50.9 (14.6)	45.8 (12.5)	43.6 (11.8)	46.8 (13.1)	41.8 (8.7)	40.2 (12.1)	55.0 (16.7)	49.0 (15.0)	46.2 (12.2)
Parental Stress Index (PSI)									
Parental Distress	39.0 (12.7)	44.8 (14.5)	46.3 (15.5)	39.0 (11.6)	43.2 (15.5)	50.5 (22.5)	39.0 (15.5)	46.0 (15.4)	43.0 (8.2)
Parent–Child Dysfunctional Interaction	44.0 (14.2)	46.4 (12.5)	45.4 (11.5)	45.8 (12.8)	46.8 (12.2)	45.5 (12.0)	42.3 (17.3)	46.2 (14.2)	45.4 (12.6)
Difficult Child	49.1 (14.8)	52.1 (13.9)	53.9 (18.2)	51.8 (14.7)	53.5 (14.7)	58.2 (23.0)	46.5 (16.6)	51.0 (14.8)	50.4 (15.1)
Total	67.1 (23.1)	70.0 (24.4)	71.1 (23.2)	61.0 (23.6)	62.8 (17.0)	67.5 (19.6)	73.2 (24.3)	75.8 (29.7)	74.0 (27.6)
Pediatric Quality of Life Family Impact Module (PedsQL FIM)									
Health-Related	58.4 (12.4)	63.8 (18.7)	64.9 (14.8)	60.3 (6.3)	70.9 (13.1)	70.3 (12.6)	56.6 (17.5)	58.0 (21.9)	60.5 (16.4)
Family	58.6 (10.3)	62.5 (21.3)	61.8 (17.7)	57.0 (5.9)	64.1 (20.2)	67.2 (15.4)	60.2 (14.3)	61.2 (24.4)	57.5 (19.8)
Total	57.5 (9.7)	63.3 (17.5)	63.0 (15.2)	58.5 (5.5)	69.1 (12.1)	68.1 (12.4)	56.4 (13.6)	58.7 (21.1)	59.0 (17.4)

Note. SD = standard deviation. Values reported as means (SD). Possible score ranges for ABC original subscales include: irritability/aggression, 0 to 45; lethargy/social withdrawal, 0 to 48; stereotypy, 0 to 21; hyperactivity, 0 to 48; and inappropriate speech, 0 to 12. Possible score ranges for ABC Fragile X subscales include: irritability/aggression, 0 to 54; socially unresponsive, 0 to 39; stereotypy, 0 to 18; hyperactivity, 0 to 30; inappropriate speech, 0 to 12; and social avoidance, 0 to 12.

**Table 4:** Effect sizes for post hoc paired *t*-tests.

Measure	Overall (*N* = 9)						DS (*N* = 4)						FXS (*N* = 5)					
	Baseline vs. Post-Treatment		Baseline vs. 12 Weeks Post-Treatment		Post-Treatment vs. 12 Weeks Post-Treatment		Baseline vs. Post-Treatment		Baseline vs. 12 Weeks Post-Treatment		Post-Treatment vs. 12 Weeks Post-Treatment		Baseline vs. Post-Treatment		Baseline vs. 12 Weeks Post-Treatment		Post-Treatment vs. 12 Weeks Post-Treatment	
	*g*	*p*	*g*	*p*	*g*	*p*	*g*	*p*	*g*	*p*	*g*	*p*	*g*	*p*	*g*	*p*	*g*	*p*
Aberrant Behavior Checklist (ABC)																		
Irritability/Aggression	0.427	0.119	0.563	0.031	0.139	0.824	2.506	0.013	1.643	0.016	−0.505	0.817	0.249	0.612	0.407	0.278	0.199	0.780
Lethargy/Social Withdrawal	0.156	0.721	0.836	0.042	0.510	0.176	0.082	0.986	0.789	0.351	0.420	0.498	0.265	0.662	0.830	0.158	0.433	0.428
Stereotypy	0.509	0.197	0.774	0.026	0.342	0.585	0.755	0.307	1.121	0.111	0.544	0.826	0.370	0.642	0.505	0.351	0.273	0.847
Hyperactivity	0.518	0.173	0.964	0.007	0.365	0.298	0.415	0.764	0.791	0.331	0.392	0.778	0.458	0.177	0.921	0.025	0.356	0.404
Inappropriate Speech	−0.020	0.997	0.410	0.397	0.417	0.386	0.214	0.929	0.812	0.369	1.104	0.628	−0.087	0.952	0.109	0.952	0.183	0.825
Aberrant Behavior Checklist (ABC)—Fragile X Subscales																		
Irritability/Aggression	-	-	-	-	-	-	-	-	-	-	-	-	0.179	0.807	0.377	0.352	0.262	0.689
Socially Unresponsive	-	-	-	-	-	-	-	-	-	-	-	-	1.460	0.032	1.153	0.044	−0.133	0.972
Stereotypy	-	-	-	-	-	-	-	-	-	-	-	-	0.375	0.663	0.454	0.464	0.175	0.934
Hyperactivity	-	-	-	-	-	-	-	-	-	-	-	-	0.536	0.044	1.178	0.003	0.412	0.168
Inappropriate Speech	-	-	-	-	-	-	-	-	-	-	-	-	−0.087	0.952	0.109	0.952	0.183	0.825
Social Avoidance	-	-	-	-	-	-	-	-	-	-	-	-	<0.001	0.999	0.595	0.539	0.388	0.539
Home Situations Questionnaire—ASD (HSQ-ASD)																		
Social Inflexibility	0.818	0.056	1.148	0.009	0.376	0.607	1.476	0.104	1.120	0.153	−0.234	0.952	0.299	0.610	0.891	0.054	0.695	0.165
Demand-Specific	0.939	0.026	0.803	0.059	−0.177	0.897	1.221	0.057	0.731	0.208	−0.522	0.594	0.428	0.536	0.713	0.316	0.225	0.873
Family Impact Questionnaire (FIQ)																		
Positive Parent	−0.009	0.980	−0.059	0.999	−0.046	0.981	0.056	0.992	−0.102	0.967	−0.190	0.929	−0.032	0.981	<0.001	0.950	0.031	0.991
Total Negative Impact	0.358	0.171	0.527	0.029	0.174	0.581	0.392	0.111	0.448	0.045	0.124	0.756	0.338	0.645	0.546	0.318	0.185	0.766
Parental Stress Index (PSI)																		
Parental Distress	−0.401	0.446	−0.489	0.275	−0.099	0.926	−0.270	0.868	−0.559	0.405	−0.326	0.675	−0.403	0.326	−0.299	0.738	0.220	0.678
Parent–Child Dysfunctional Interaction	−0.174	0.576	−0.107	0.983	0.079	0.660	−0.070	0.818	0.018	0.987	0.090	0.735	−0.225	0.741	−0.189	0.940	0.054	0.894
Difficult Child	−0.198	0.851	−0.271	0.551	−0.105	0.853	−0.104	0.967	−0.293	0.647	−0.214	0.786	−0.256	0.823	−0.220	0.930	0.036	0.964
Total	−0.115	0.409	−0.163	0.262	−0.044	0.940	−0.074	0.955	−0.261	0.559	−0.225	0.723	−0.082	0.270	−0.025	0.455	0.057	0.885
Pediatric Quality of Life Family Impact Module (PedsQL FIM)																		
Health-Related	−0.314	0.315	−0.444	0.208	−0.063	0.955	−0.899	0.369	−0.872	0.407	0.042	0.996	−0.064	0.908	−0.208	0.603	−0.117	0.812
Family	−0.218	0.940	−0.208	0.978	0.034	0.989	−0.411	0.663	−0.756	0.449	−0.151	0.918	−0.047	0.716	0.134	0.370	0.152	0.767
Total	−0.388	0.344	−0.410	0.384	0.018	0.996	−0.983	0.275	−0.864	0.337	0.074	0.985	−0.113	0.996	−0.146	0.989	−0.013	0.998

## Data Availability

The raw data supporting the conclusions of this article will be made available by the authors on request.

## Abbreviations

The following abbreviations are used in this manuscript:

FXS	Fragile X Syndrome
DS	Down Syndrome
RUBI	Research Units in Behavioral Intervention
RUBI-DD	Research Units in Behavioral Intervention for non-autistic developmental disabilities
ID	Intellectual Disability
ASD	Autism Spectrum Disorder
ADHD	Attention Deficit Hyperactivity Disorder
ABA	Applied Behavior Analysis
ABC	Aberrant Behavior Checklist
IQ	Intelligence Quotient
CGI	Clinical Global Impressions
PSI	Parenting Stress Inventory
HSQ	Home Situations Questionnaire
FIQ	Family Impact Questionnaire
PCSQ	Parent’s Consumer Satisfaction Questionnaire
PedsQL FIM	Pediatric Quality of Life Family Impact Module
